# Evaluation of bone mineral density in adolescent idiopathic scoliosis using a three-dimensional finite element model: a retrospective study

**DOI:** 10.1186/s13018-023-04413-0

**Published:** 2023-12-07

**Authors:** Chaofan Han, Chaochao Zhou, Hanwen Zhang, Peng Yin, Runsheng Guo, Wei Wang, Yiqi Zhang, Thomas Cha, Guoan Li, Yong Hai

**Affiliations:** 1 Department of Orthopedic Surgery, Beijing Chaoyang Hospital, Capital Medical University, Beijing, China; 2 Department of Radiology, Feinberg School of Medicine, Northwestern University, Chicago, IL USA; 3 First Affiliated Hospital of Nanchang University, Jiangxi, China; 4 Beihang University, Beijing, China; 5 Orthopaedic Spine Center, Massachusetts General Hospital, Harvard Medical School, Boston, USA; 6 Department of Orthopaedic Surgery, Bioengineering Research Center, NewtonWellesley Hospital and Harvard Medical School, Newton, USA

**Keywords:** Adolescent idiopathic scoliosis, Bone mineral density, Computed tomography, Finite element model, Osteoporosis

## Abstract

**Background:**

Adolescent idiopathic scoliosis (AIS) is often accompanied by osteopenia and osteoporosis, which can cause serious complications. The aim of this study was to determine the specific bone mineral density (BMD) of each vertebral body in patients with AIS using biomechanical finite element modeling based on three-dimensional (3D) reconstruction.

**Methods:**

This retrospective study involved 56 patients with AIS. Computed tomography (CT) and radiography were performed. Spinal vertebrae were segmented from the spinal CT images of patients with AIS to reconstruct 3D vertebral models. The vertebral models were meshed into tetrahedral finite elements to assess the BMD.

**Results:**

The mean main curve Cobb angle was 88.6 ± 36.7°, and the mean kyphosis angle was 36.8 ± 31.5°. The mean BMD of the global spine was 0.83 ± 0.15 g/cm^2^. The highest BMD was measured on the concave side of the apex (0.98 ± 0.16 g/cm^2^). Apical vertebral BMD was negatively correlated with age and height (*r* = − 0.490, *p* = 0.009 and *r* =  − 0.478, *p* = 0.043, respectively). There were no significant differences in BMD values between the concave and convex sides (*p* > 0.05).

**Conclusions:**

The 3D finite element modeling of BMD in patients with AIS is a reliable and accurate BMD measurement method. Using this method, the overall BMD of patients with AIS was shown to gradually decrease from the top to the bottom of the spine. Our findings provide valuable insights for surgical planning, choice of screw trajectories, and additional biomechanical analyzes using finite element models in the context of scoliosis.

## Background

Adolescent idiopathic scoliosis (AIS) is a complex, three-dimensional (3D) spinal deformity of unknown etiology [1]. Patients with scoliosis often have osteopenia and osteoporosis [2–4]. Currently, the conventional method for measuring bone mineral density (BMD) is dual-energy X-ray absorptiometry (DXA) [5]; however, this method can only be used to measure the density of the distal radius, lower lumbar vertebrae, and femur [6]. The density of the thoracic vertebrae cannot be measured using DXA owing to rib occlusion or unclear imaging findings [7]. Clear images of the thoracic vertebrae are particularly difficult to obtain in patients with scoliosis. Some scholars have attempted to convert the Hounsfield unit (HU) value of the computed tomography (CT) section image to the BMD value [8, 9]; however, the results were too variable to be of clinical significance.

Surgical treatment of scoliosis is highly dependent on pedicle screws [10]. Reduced bone mass can lead to a decrease in the holding and pull-out forces of pedicle screws, resulting in screw loosening and serious complications [11]. Therefore, it is vital to obtain accurate spinal BMD measurements in patients with scoliosis.

Biomechanical finite element modeling based on 3D reconstruction may be used to accurately measure the specific BMD of each vertebral body [12]. Thus, herein, we aimed to used biomechanical finite element modeling to measure the true BMD value of the concave and convex sides of each vertebral body in patients with AIS. We selected a specific plane for cutting the reconstructed model, and divided the cut plane into two regions of interest (ROIs) on the concave and convex sides. The obtained ROI was analyzed to extract pixels and determine the BMD value. This method facilitates observation of the changing trend of BMD on the concave or convex side, as well as the correlation between the curvature magnitude and type.

## Materials and methods

This retrospective study was approved by the Institutional Review Board of Beijing Chaoyang Hospital, and informed consent was obtained from all patients. The data of 56 patients diagnosed with AIS between December 2015 and December 2018 were retrospectively reviewed. The inclusion criteria were as follows: (1) patients with Lenke type I or II scoliosis involving apical vertebrae from T8–T10; (2) those who had a complete preoperative 3D CT DICOM format file; (3) those with Risser grade IV or V scoliosis; (4) those with preoperative whole-spine standing position X-ray films; and (5) those for whom the threshold selection was clear when reconstructing the 3D model. The exclusion criteria were as follows: patients with (1) bone metabolism-related diseases, such as thyroid dysfunction, and (2) neurofibromatosis combined with scoliosis or other scoliotic diseases involving BMD changes. The extracted demographic data included sex, age, and body mass index (BMI).

### Radiographic parameters

Standing, full-length, spinal coronal and sagittal radiographs were obtained by two senior spinal surgeons. The radiographic parameters included the Cobb angle, kyphosis angle, apical vertebral translation (AVT), coronal balance, and Risser sign.

### Mapping and measurements of bone density from CT data

**Fig. 1:**
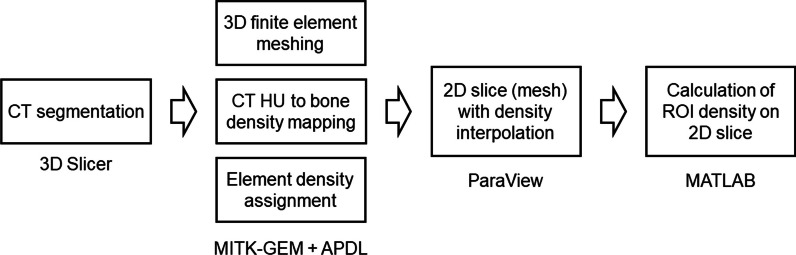
A flow chart to summarize the steps of bone density mapping and measurement. The software packages adopted in the procedure are also listed below each step

A flowchart of the procedure for bone density mapping and measurements from CT data is shown in Fig. 1. First, 3D geometrical vertebral models were segmented from CT data. Then, 3D finite element vertebral models were created undergoing pre-processing steps, including finite element meshing, bone density mapping from CT Hounsfield unit (HU) values, and assignment of bone density to each finite element. Subsequently, 2D slicing of the 3D finite element vertebral models along a plane across pedicles was performed, resulting in 2D surface meshes, where node densities were determined by interpolating 3D finite element densities. Finally, the densities of ROIs at the concave and convex sides of the 2D slice were calculated. The details of the processing are further described as follows.

The patient’s CT image (DICOM file) was imported to the 3D Slicer software (open source, V.4.10.1; https://www.slicer.org/), and the T-spine 1.0 B30 f sequence was read to visualize the coronal, sagittal, and surrounding 3D CT images of the thoracolumbar vertebrae. We reconstructed the 3D model from segments T1 to L5 for complete reconstruction. Subsequently, the entire reconstructed model spine was cut parallel to the endplate at the intervertebral disk plane in segments, and 17 thoracolumbar vertebrae were individually segmented for finite element modeling.

**Fig. 2:**
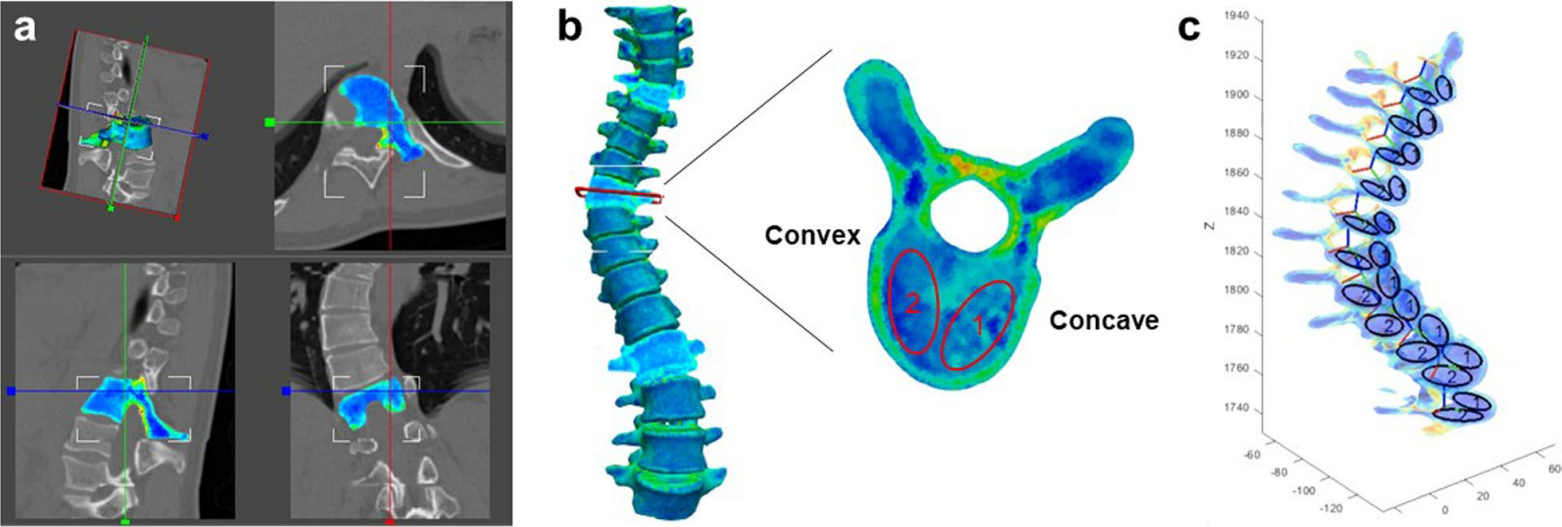
Procedure for bone density mapping and measurement. **a** Mapping bone density from CT. **b** Slicing a vertebra and aligning ROIs on the concave (labeled “1”) and convex (labeled “2”) sides to calculate mean densities. **c** Visualizing ROIs on vertebral slices in the entire spine to ensure that their concave and convex labels are consistent. CT: computed tomography; ROI: region of interest

Spinal vertebrae were segmented from the spine CT images of patients with scoliosis to reconstruct 3D vertebral models using the 3D Slicer software. These models were then imported into MITK-GEM (https://araex.github.io/mitk-gem-site/) and meshed into tetrahedral finite elements with a mean size of 1 mm (same as the spatial resolution of our CT scanner). Each finite element was assigned an apparent bone density value derived from the CT images using ANSYS APDL scripts (V15.0 ANSYS, Inc., Canonsburg, PA, USA; based on a template APDL script provided by MITK-GEM) [12, 13], as shown in Fig. 2a. We adopted well-established relationships [12, 13] or mapped the bone density from the CT images using Eqs. 1–3:

1 $$\rho_{{{\text{HA}}}} = 0.0026HU - 0.0829$$

2 $$\rho_{{{\text{ash}}}} = \left( {\rho_{{{\text{HA}}}} + 0.09} \right)/1.14$$

3 $$\rho_{{{\text{app}}}} = \rho_{{{\text{ash}}}} /0.6$$

where ${\text{HU}}$ is the Hounsfield unit (HU) value of spine CT, $\rho_{{{\text{HA}}}}$ is the hydroxyapatite density, $\rho_{{{\text{ash}}}}$ is the ash density, and $\rho_{{{\text{app}}}}$ is the apparent density assigned to the finite elements of the vertebral models.

Once a 3D spine model with bone density distribution was obtained, it was imported into ParaView (https://www.paraview.org/) to slice each vertebra by aligning a slicing plane across both pedicles, while the bone density was interpolated onto the slice (Fig. 2b). Furthermore, a custom MATLAB program (MathWorks, Natick, MA, USA) was developed to calculate the mean density in an elliptical ROI on a bone density slice (Fig. 2b). The size and shape of the ROI could be adjusted to align two identical ROIs on the concave and convex sides simultaneously. This allowed us to investigate the bone densities both on the concave and convex sides of the vertebral cross-sections along the complicated scoliotic spine curve (Fig. 2c).

**Fig. 3:**
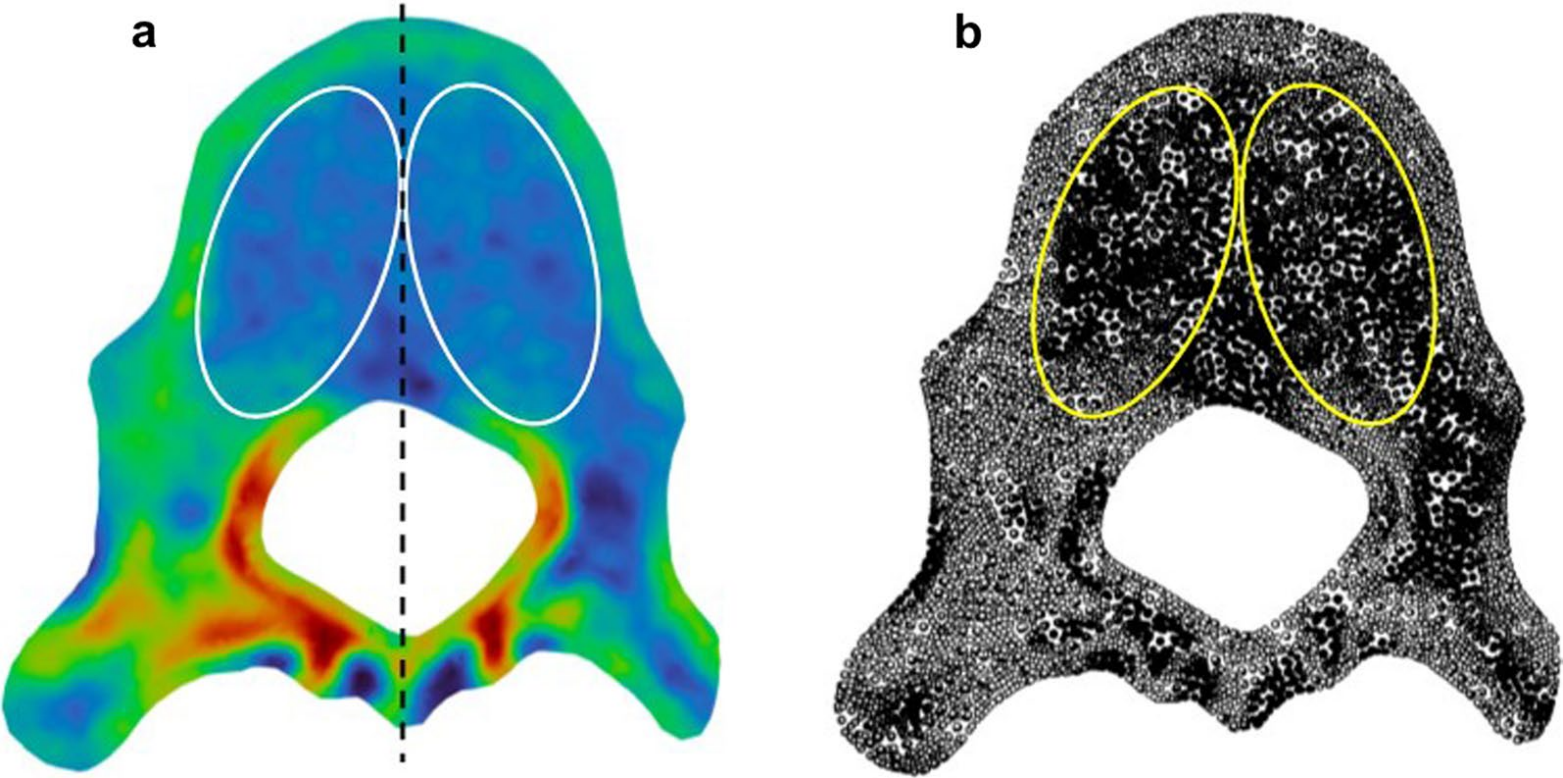
**a** Definition of ROIs on a 2D slice (note: densities are presented in a rainbow plot) and **b** calculation of ROI densities by node densities of the 2D surface (slice) mesh (note that solid and hollow nodes indicate high and low densities, respectively)

In particular, we defined four ROIs on the slice of each vertebra to measure the vertebral and pedicle bone density at both the concave and convex sides (Fig. 3a). For the vertebral ROIs, each of them is tangent to the middle line of the vertebral cross-section, as well as the internal cortical shells of the vertebral body and the spinal canal. Furthermore, as shown in Fig. 3b, the slicing of the 3D finite element vertebral model resulted in a 2D surface mesh consisting of dense nodes with interpolated densities. Consequently, the density of a ROI is represented by the average density of the nodes within the ROI.

### Statistical methods

Data are reported as mean ± standard deviation unless otherwise specified. A paired t-test was used to compare the differences in BMD between the DXA method and 3D finite element method at different positions and between the concave and convex sides at different positions. An independent samples t-test was used to compare the differences in BMD at different positions on the same side. Pearson’s correlation coefficient was used to assess the association between BMD changes and other factors, such as age, sex, Risser sign, and main curve Cobb angle. Differences were analyzed using two-tailed paired Student’s t-tests, and correlations were analyzed using Spearman’s rank test. The statistical analyzes were performed using SPSS version 25.0 (IBM Corp., Armonk, NY, USA). Statistical significance was set at *p* < 0.05.

## Results

Fifty-six patients with Lenke type I or II scoliosis were recruited. Table 1 presents the demographic and radiographic data of the patients.

**Table 1:** Demographic and radiographic data of the patients

Parameter	Value
Total number of patients	56
Sex, male:female	12:44
Age, years	19.40 (5.94)
Height, cm	148.90 (16.24)
Weight, kg	44.20 (12.12)
BMI, kg/m^2^	19.70 (3.21)
Risser sign	4.77 (0.42)
Main Cobb angle	88.6° (36.7°)
Kyphosis angle	36.8° (31.5°)
AVT, mm	68.3 (6.5)
Coronal balance	18.2 (6.5)

BMI: body mass index; AVT: apical vertebral translation. Data are reported as mean (standard deviation) unless otherwise specified

### 3D reconstruction analysis results

The BMD measured using DXA was slightly higher than that measured using the 3D finite element method at L1–L5 respectively, although the difference was not statistically significant (Table 2). The BMD of the whole spine was 0.83 ± 0.15 g/cm^2^ and that of the concave and convex sides was 0.84 ± 0.16 and 0.83 ± 0.15 g/cm^2^, respectively, with no significant differences between the sides (*p* = 0.589). The BMD of the apical vertebra was significantly higher than that of the whole spine, reaching 0.86 ± 0.13 g/cm^2^. Furthermore, spinal BMD was highest at the concave side of the apical vertebra, reaching 0.98 ± 0.16 g/cm^2^. In the main curve range (U/L-EV), the BMD of the concave side was significantly greater than that of the convex side (*p* < 0.001).

**Table 2:** Mean BMD (g/cm^2^) of the lumbar spine measured using DXA and 3D finite element modeling

	L1	L2	L3	L4	L5
DXA	0.7500 ± 0.0943	0.7580 ± 0.1009	0.7565 ± 0.1126	0.7549 ± 0.1026	0.8157 ± 0.1098
3D finite element	0.7370 ± 0.109	0.7434 ± 0.115	0.7312 ± 0.111	0.7519 ± 0.125	0.8189 ± 0.164
*p*-value	0.1376	0.5735	0.8725	0.3518	0.0081

BMD: bone mineral density

**Fig. 4:**
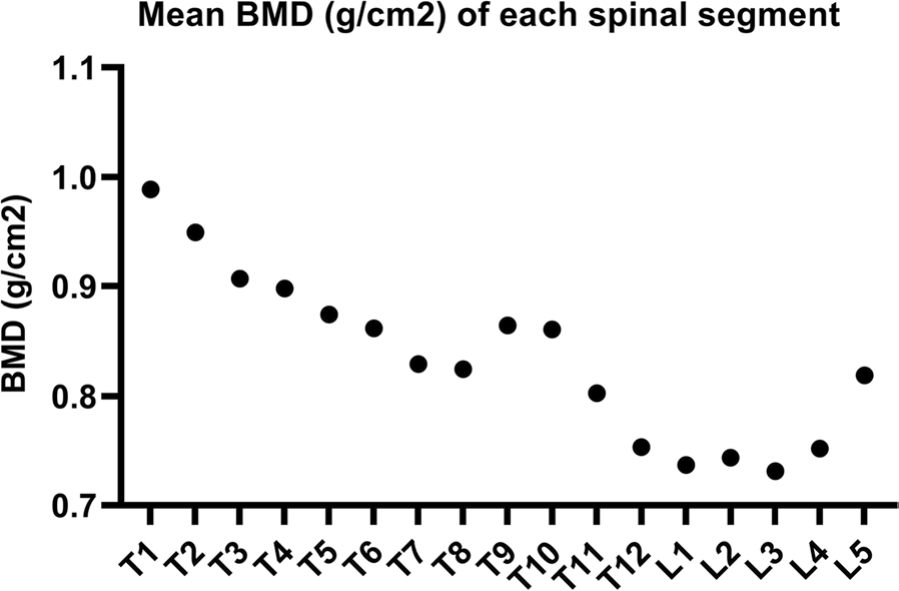
Mean bone mineral density (g/cm^2^) of each spinal segment

**Fig. 5:**
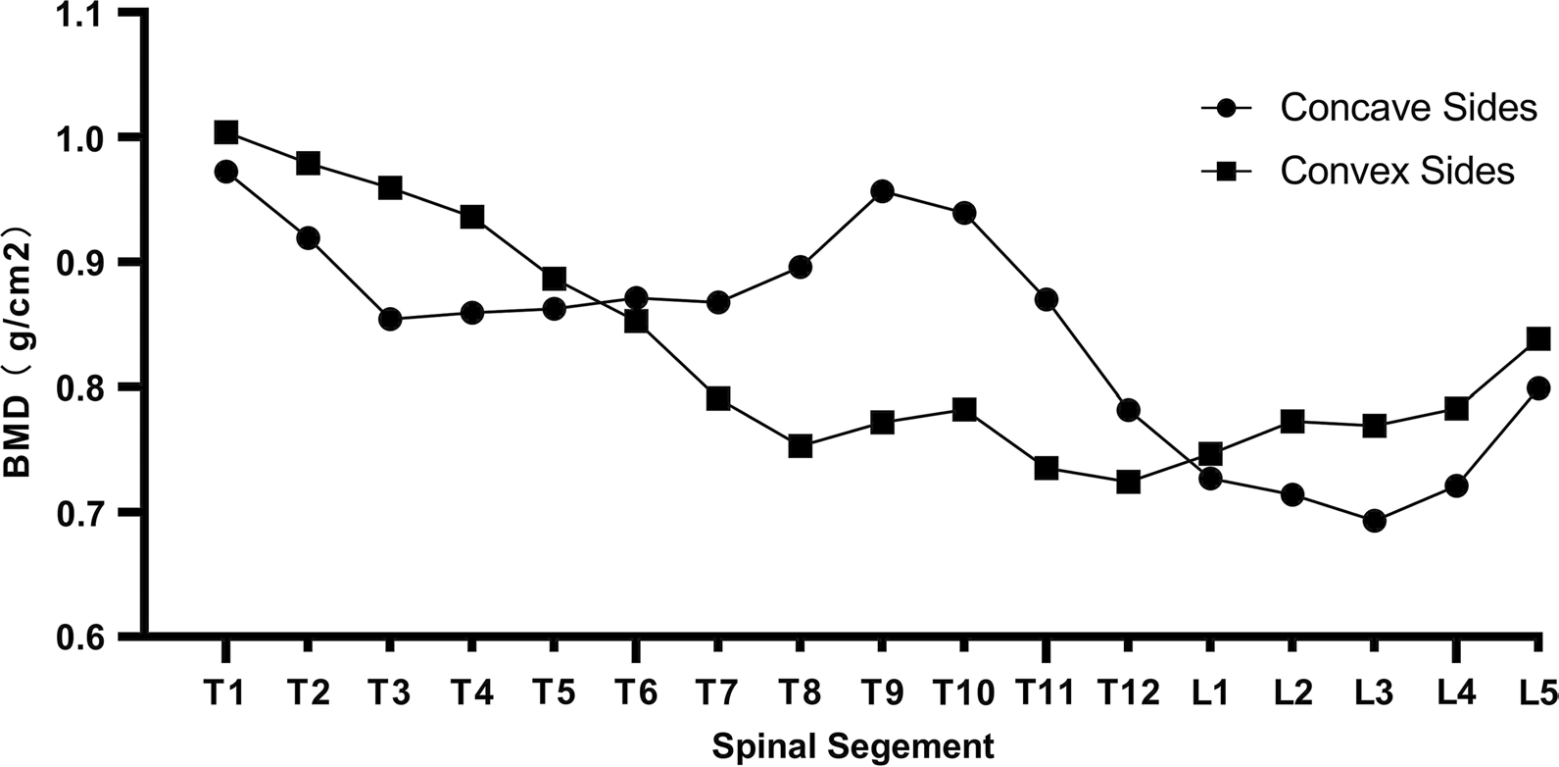
Bone mineral densities on the concave and convex sides. Overall, they gradually decrease from the cranial to the caudal vertebrae of the spine, and the change trends of both sides are opposing. BMD: bone mineral density

The BMD of each spinal segment is shown in Fig. 4. The highest BMD was at T1, the lowest at L3, and the intermediate values occurred at T9 and T10. According to our data, BMD gradually decreased from top to bottom, while the change trends on the concave and convex sides were opposite to each other (Fig. 5). Apical vertebral BMD was negatively correlated with age and height (*r* =  − 0.490, *p* = 0.009 and *r* = − 0.478, *p* = 0.043, respectively). The main curve Cobb angle was moderately correlated with the kyphosis angle (*r* = 0.436, *p* = 0.020 and *r* = 0.465, *p* = 0.013, respectively). No significant correlations were observed between the other factors and changes in BMD (Table 3).

**Table 3:** Pearson’s correlation analysis of the correlation between BMD changes and other factors

Factors	Bilateral sides	Concave sides	Convex sides	Apical vertebra	Main curve range (U/L-EV)
Mean age	*r* = 0.123, *p* = 0.540	*r* = 0.118, *p* = 0.559	*r* = 0.129, *p* = 0.520	***r* = − 0.490, *p* = 0.009**	*r* = 0.317, *p* = 0.107
Mean height	*r* = 0.097, *p* = 0.631	*r* = 0.044, *p* = 0.828	*r* = 0.147, *p* = 0.464	***r* = − 0.478, *p* = 0.042**	*r* = 0.000, *p* = 0.999
Mean weight	*r* = 0.092, *p* = 0.648	*r* = 0.025, *p* = 0.903	*r* = 0.158, *p* = 0.430	*r* = 0.374, *p* = 0.055	*r* = 0.030, *p* = 0.882
BMI	*r* = 0.020, *p* = 0.922	*r* = 0.027, *p* = 0.894	*r* = 0.069, *p* = 0.733	*r* = 0.167, *p* = 0.405	*r* = 0.055, *p* = 0.787
Risser sign	*r* = 0.081, *p* = 0.688	*r* = 0.074, *p* = 0.714	*r* = 0.094, *p* = 0.641	*r* = 0.037, *p* = 0.856	*r* = 0.058, *p* = 0.775
Main Cobb angle	*r* = 0.020, *p* = 0.921	*r* = 0.061, *p* = 0.758	*r* = 0.035, *p* = 0.860	***r* = 0.436, *p* = 0.020**	*r* = 0.190, *p* = 0.333
Kyphosis angle	*r* = 0.033, *p* = 0.866	*r* = 0.140, *p* = 0.477	*r* = 0.079, *p* = 0.690	***r* = 0.465, *p* = 0.013**	*r* = 0.230, *p* = 0.239
AVT	*r* = 0.092, *p* = 0.640	*r* = 0.040, *p* = 0.842	*r* = 0.142, *p* = 0.472	*r* = 0.236, *p* = 0.226	*r* = 0.017, *p* = 0.930
Coronal balance	*r* = 0.093, *p* = 0.636	*r* = 0.061, *p* = 0.758	*r* = 0.126, *p* = 0.523	*r* = 0.263, *p* = 0.176	*r* = 0.017, *p* = 0.932

Bold value indicates significance threshold was set at 5% (*p* < 0.05)

BMD: bone mineral density; BMI: body mass index; AVT: apical vertebral translation

## Discussion

In this study, a 3D finite element model of the whole spine and 3D CT reconstruction were used to determine the density of the vertebral body. Accurate BMD values of patients with AIS were obtained by reading the color values in the ROI. We found that the overall BMD of patients with scoliosis gradually decreases from top to bottom. The mean BMD of the concave side is slightly higher than that of the convex side. The BMD of the apical vertebra is the largest, and the mean value increases concurrent with the increase of the bending angle. The trend of change in the BMD of the concave side was opposite to that of the convex side.

Patients with scoliosis often exhibit decreased bone mass and osteoporosis [2–4, 14]. The BMD is directly related to the severity of adolescent scoliosis [15] and represents the load-bearing capacity of bone [16]. Therefore, BMD is a particularly important factor in the surgical treatment of patients with scoliosis. Currently, the gold-standard method for measuring BMD is DXA [5]. Cook et al. [14] compared the BMD of 44 female children with AIS and 44 healthy female children. The mean BMD of the femoral neck and lumbar spine in patients with AIS were 0.93 and 1.01 g/cm^2^, respectively, which were significantly lower than that of healthy children. Snyder et al. [17] conducted a prospective study, involving 52 women with AIS, on the relationship between BMD and brace treatment. The results showed that the mean BMD was 0.848 g/cm^2^ for L1–L4 and 0.776 g/cm^2^ for the femoral neck.

In our study, the mean BMD of L2–L4 was 0.742 g/cm^2^, which was slightly lower than that reported previously [14, 17–20]. There are several possible reasons for these diskrepancies. First, the measurement methods used in the studies are different. The DXA method calculates BMD based on positive radiography according to the imaging value, which is a two-dimensional numerical value [21, 22]. The present study applied a 3D model cutting-entity analysis. Second, when measuring BMD by two-dimensional radiography, the coronal images contain multiple structures in the three-column structure of the spine, including the cortical bone of the anterior column, cancellous bone of the middle column, and posterior complex of the posterior column. As the density of the cortical bone and posterior complex is significantly higher than that of the cancellous bone, DXA measures BMD without differentiating between cancellous and cortical bone; thus, BMD values obtained using this method are expectantly higher than those of pure cancellous bone [23]. In addition, if the anterior cortical bone has significant calcification due to severe scoliosis, this will also significantly increase the BMD values obtained using DXA. Third, two-dimensional imaging is a horizontal image acquisition technique parallel to the ground and lacks consideration for alignment factors, such as lumbar lordosis and vertebral body rotation [24].

Some researchers have measured BMD using CT image HUs [21, 23, 25], which is rapid, simple, and reproducible method wherein results are unaffected by severe scoliosis, surrounding osteophytes, and vascular calcifications [21]. There was a consensus that HU values > 160 indicate a significantly reduced risk of osteoporosis, whereas HU values < 110 were significantly correlated with osteoporosis [25]. The measurement of BMD using CT images has improved in accuracy compared with the DXA method, but some drawbacks remain [25]. First, the selection of CT images is determined by the imaging technician, and the mid-axial section may not accurately represent bone quality [23]. Second, the measurement of the HU value was considered to be strongly correlated with the results measured using DXA, and the sensitivity and specificity of the HU were high [21, 26, 27]. However, HU values in some studies still differ from the results obtained using DXA [23, 28]. Therefore, the HU value can be used as a prompt standard to determine whether patients have bone mass reduction or osteoporosis [29], but it cannot be used as an accurate assessor of bone density or a reference value for accurate basic research or 3D finite element modeling.

By comparing the results of the current study to those of previous studies and analyzing the reasons for differences, it became apparent that the reconstruction of a 3D finite element model of scoliosis to analyze the specific values of BMD is an accurate and reliable measurement method. First, 3D finite element modeling based on CT image data enables the measurements of bone density on the cross sections of vertebral bodies with specific orientations (across the pedicles). Second, in this study, we purposefully selected specific planes to overcome the limitation of unclear objectives in the DXA and CT image measurements. Third, in 3D finite element modeling, the ROI in a specific plane can be selected such that BMD values can be measured and compared, especially for pedicle screw placement. In this study, BMD measurements were performed in a specific plane that simulated pedicle screw placement because BMD is positively correlated with the holding and pull-out forces of internal fixation. Determination of osteoporosis prior to spinal fusion is critical because osteoporosis is a risk factor for pseudarthrosis owing to the higher risk of screw loosening [30]; the higher the BMD, the stronger the internal fixation.

**Fig. 6:**
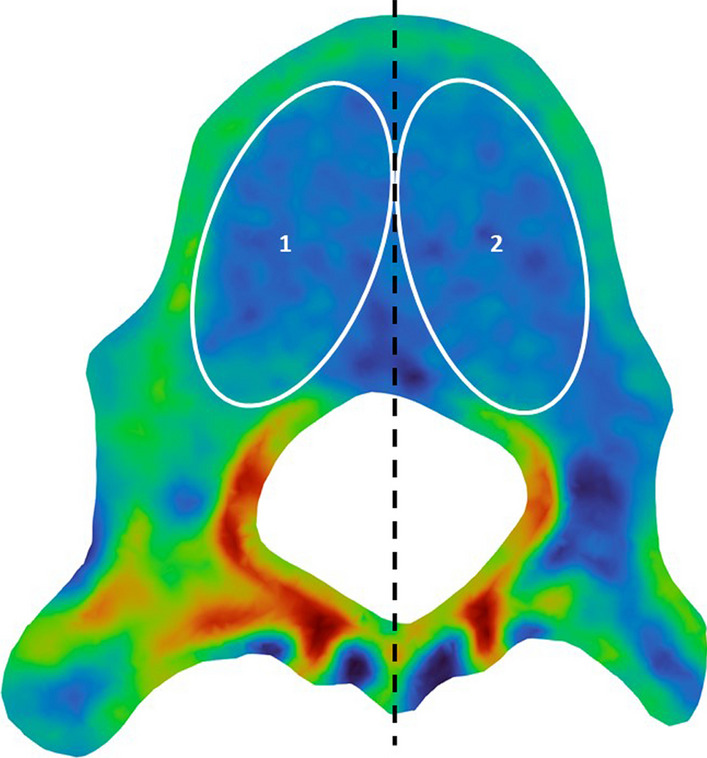
The bone densities of ROIs on a bone density slice. Region 1 is the concave side ROI, and region 2 is convex side ROI. A high-density zone on the inner side of the region 1 is the 'high-density zone' of the bone mineral density value. ROI: region of interest

Therefore, the results of different scoliosis segments are helpful for the selection of internal fixation materials and sizes in clinical treatment, future internal fixation devices [31, 32], and spinal surgery improvement [33, 34]. At present, most spinal surgeons still manually place pedicle screws, and the placement direction depends on the surgeon’s experience. According to our results, the BMD on the concave side was not uniform and greater than that on the convex side. Many vertebral bodies have a high-density zone in the cancellous bone of the concave side (Fig. 6), and the density of some high-density zones is close to that of the lateral wall of the pedicle and cortical bone of the vertebral body. If the screw placement route passes through this “high-density belt,” the maximum holding and pull-out force values of the screw can be achieved. In the past, we have routinely selected the screw diameter in relation to the pedicle diameter; however, according to the results of this study (gradual reduction of BMD from top to bottom of the spine in patients with scoliosis), we can now select the screw diameter according to the BMD.

As the 3D changes observed in deformed spines are obvious and individual differences are large, the dearth of studies in the field of biomechanical research on scoliosis remains extensive. Measuring the BMD using 3D finite element technology has great significance for future research, as BMD is the basis of all bone studies.

This study has some limitations. First, the sample size was insufficient, and BMD assessments should involve a census of a large sample size to represent the situation of a group of people. Therefore, future studies should expand the sample size and conduct a detailed grouping of women before menarche, after menarche, and after adulthood. This study focused on identifying the relative difference of the bone densities of the vertebrae on the concave and convex sides. The relative difference of bone densities has high confidence, as it is directly related with the difference in the HU values of CT data (see the linear mapping in Eqs. 1–3). However, in future work, it is necessary to further evaluate and improve the accuracy of the magnitude of bone density derived from CT HU values, by comparing the density of a phantom and calibrating bone density mapping parameters. Finally, all model reconstructions and finite element modeling were manually performed, so certain errors inevitably existed in the operations (e.g., CT segmentation and ROI placement), and future work is needed to further investigate the repeatability of the finite element reconstruction process.

## Conclusions

In this study, we found, using 3D finite element modeling, that the BMD of the whole spine was 0.83 ± 0.15 g/cm^2^, with no significant difference between the convex and concave sides. The overall BMD of patients with AIS gradually decreased from the top to the bottom of the spine. Thus, 3D finite element modeling of BMD in patients with scoliosis is a reliable and accurate measurement method that provides a theoretical basis for the future improvement of spinal surgical internal fixation devices and biological analysis of scoliosis. The results of our study will be helpful for surgical planning, screw trajectory selection, and further finite element biomechanical analysis of scoliosis.

## Data Availability

The datasets used and/or analyzed during the current study are available from the corresponding author on reasonable request.

## Abbreviations

3D: Three-dimensional
AIS: Adolescent idiopathic scoliosis
AVT: Apical vertebral translation
BMD: Bone mineral density
BMI: Body mass index
CT: Computed tomography
DXA: Dual-energy X-ray absorptiometry
HU: Hounsfield unit
ROI: Region of interest

## References

[1] Weinstein SL, Dolan LA, Cheng JC, Danielsson A, Morcuende JA (2008). Adolescent idiopathic scoliosis. Lancet.

[2] Cheng JC, Guo X, Sher AH (1999). Persistent osteopenia in adolescent idiopathic scoliosis A longitudinal follow up study. Spine (Phila Pa 1976).

[3] Li XF, Li H, Liu ZD, Dai LY (2008). Low bone mineral status in adolescent idiopathic scoliosis. Eur Spine J.

[4] Diarbakerli E, Savvides P, Wihlborg A, Abbott A, Bergstrom I, Gerdhem P (2020). Bone health in adolescents with idiopathic scoliosis. Bone Joint J.

[5] Mazess RB, Barden HS, Bisek JP, Hanson J (1990). Dual-energy x-ray absorptiometry for total-body and regional bone-mineral and soft-tissue composition. Am J Clin Nutr.

[6] Crabtree NJ, Arabi A, Bachrach LK, Fewtrell M, El-Hajj Fuleihan G, Kecskemethy HH, Jaworski M, Gordon CM (2014). International Society for Clinical D: Dual-energy X-ray absorptiometry interpretation and reporting in children and adolescents: the revised 2013 ISCD Pediatric Official Positions. J Clin Densitom.

[7] Briggs AM, Wark JD, Kantor S, Fazzalari NL, Greig AM, Bennell KL (2006). Bone mineral density distribution in thoracic and lumbar vertebrae: An ex vivo study using dual energy X-ray absorptiometry. Bone.

[8] Mai HT, Mitchell SM, Hashmi SZ, Jenkins TJ, Patel AA, Hsu WK (2016). Differences in bone mineral density of fixation points between lumbar cortical and traditional pedicle screws. Spine J.

[9] Zaidi Q, Danisa OA, Cheng W (2019). Measurement techniques and utility of hounsfield unit values for assessment of bone quality prior to spinal instrumentation a review of current literature. Spine.

[10] Min K, Waelchli B, Hahn F (2005). Primary thoracoplasty and pedicle screw instrumentation in thoracic idiopathic scoliosis. Eur Spine J.

[11] Di Silvestre M, Parisini P, Lolli F, Bakaloudis G (2007). Complications of thoracic pedicle screws in scoliosis treatment. Spine (Phila Pa 1976).

[12] Pauchard Y, Fitze T, Browarnik D, Eskandari A, Pauchard I, Enns-Bray W, Palsson H, Sigurdsson S, Ferguson SJ, Harris TB (2016). Interactive graph-cut segmentation for fast creation of finite element models from clinical ct data for hip fracture prediction. Comput Methods Biomech Biomed Engin.

[13] Zhou C, Jin S, Willing R (2016). Simulation of extracellular matrix remodeling by fibroblast cells in soft three-dimensional bioresorbable scaffolds. Biomech Model Mechanobiol.

[14] Cook SD, Harding AF, Morgan EL, Nicholson RJ, Thomas KA, Whitecloud TS, Ratner ES (1987). Trabecular bone mineral density in idiopathic scoliosis. J Pediatr Orthop.

[15] Lee WT, Cheung CS, Tse YK, Guo X, Qin L, Lam TP, Ng BK, Cheng JC (2005). Association of osteopenia with curve severity in adolescent idiopathic scoliosis: a study of 919 girls. Osteoporos Int.

[16] Prakash, Prabhu LV, Saralaya VV, Pai MM, Ranade AV, Singh G, Madhyastha S: Vertebral body integrity: a review of various anatomical factors involved in the lumbar region. Osteoporos Int 2007, **18**(7):891–903. 10.1007/s00198-007-0373-5 17404781

[17] Snyder BD, Katz DA, Myers ER, Breitenbach MA, Emans JB (2005). Bone density accumulation is not affected by brace treatment of idiopathic scoliosis in adolescent girls. J Pediatr Orthop.

[18] Cheuk KY, Hu Y, Tam EMS, Shi L, Yu FWP, Hung VWY, Lai KCY, Cheng WHW, Yip BHK, Qin L (2019). Bone measurements at multiple skeletal sites in adolescent idiopathic scoliosis-an in vivo correlation study using DXA, HR-pQCT and QCT. Arch Osteoporos.

[19] Cheng JC, Qin L, Cheung CS, Sher AH, Lee KM, Ng SW, Guo X (2000). Generalized low areal and volumetric bone mineral density in adolescent idiopathic scoliosis. J Bone Miner Res.

[20] Lam TP, Hung VW, Yeung HY, Tse YK, Chu WC, Ng BK, Lee KM, Qin L, Cheng JC (2011). Abnormal bone quality in adolescent idiopathic scoliosis: a case-control study on 635 subjects and 269 normal controls with bone densitometry and quantitative ultrasound. Spine (Phila Pa 1976).

[21] Zaidi Q, Danisa OA, Cheng W (2019). Measurement techniques and utility of hounsfield unit values for assessment of bone quality prior to spinal instrumentation. Spine.

[22] Kirmani S, Christen D, van Lenthe GH, Fischer PR, Bouxsein ML, McCready LK, Melton LJ, Riggs BL, Amin S, Muller R (2009). Bone structure at the distal radius during adolescent growth. J Bone Miner Res.

[23] Choi MK, Kim SM, Lim JK (2016). Diagnostic efficacy of Hounsfield units in spine CT for the assessment of real bone mineral density of degenerative spine: correlation study between T-scores determined by DEXA scan and Hounsfield units from CT. Acta Neurochir (Wien).

[24] Pappou IP, Girardi FP, Sandhu HS, Parvataneni HK, Cammisa FP, Schneider R, Frelinghuysen P, Lane JM (2006). diskordantly high spinal bone mineral density values in patients with adult lumbar scoliosis. Spine (Phila Pa 1976).

[25] Deshpande N, Hadi MS, Lillard JC, Passias PG, Linzey JR, Saadeh YS, LaBagnara M, Park P (2023). Alternatives to DEXA for the assessment of bone density: a systematic review of the literature and future recommendations. J Neurosurg Spine.

[26] Smith A, Khan M, Varney E, Liu B, Roda M, Reed C, Morris R, Joyner D, Lirette ST, Mosley T (2019). Opportunistic bone density screening for the abdominal radiologist using colored CT images: a pilot retrospective study. Abdom Radiol (NY).

[27] Wagner SC, Formby PM, Helgeson MD, Kang DG (2016). Diagnosing the undiagnosed: osteoporosis in patients undergoing lumbar fusion. Spine (Phila Pa 1976).

[28] Kim K, Song SH, Kim IJ, Jeon YK (2021). Is dual-energy absorptiometry accurate in the assessment of bone status of patients with chronic kidney disease?. Osteoporos Int.

[29] Kim KH, Kim TH, Kim SW, Kim JH, Lee HS, Chang IB, Song JH, Hong YK, Oh JK (2022). Significance of measuring lumbar spine 3-dimensional computed tomography hounsfield units to predict screw loosening. World Neurosurg.

[30] Okuyama K, Abe E, Suzuki T, Tamura Y, Chiba M, Sato K (2001). Influence of bone mineral density on pedicle screw fixation: a study of pedicle screw fixation augmenting posterior lumbar interbody fusion in elderly patients. Spine J.

[31] Ishikawa K, Toyone T, Shirahata T, Kudo Y, Matsuoka A, Maruyama H, Hayakawa C, Tani S, Sekimizu M, Tsuchiya K (2018). A novel method for the prediction of the pedicle screw stability: regional bone mineral density around the screw. Clin Spine Surg.

[32] Zhang R, Gao H, Li H, Xing T, Jia C, Zhang J, Dong F, Shen C (2019). Differences in bone mineral density of trajectory between lumbar cortical and traditional pedicle screws. J Orthop Surg Res.

[33] Yagi M, Fujita N, Tsuji O, Nagoshi N, Asazuma T, Ishii K, Nakamura M, Matsumoto M, Watanabe K (2018). Low bone-mineral density is a significant risk for proximal junctional failure after surgical correction of adult spinal deformity: a propensity score-matched analysis. Spine (Phila Pa 1976).

[34] Yeh YC, Niu CC, Chen LH, Chen WJ, Lai PL (2019). The correlations between the anchor density and the curve correction of adolescent idiopathic scoliosis surgery. BMC Musculoskelet Disord.

